# Determination of Residual Nonsteroidal Anti-Inflammatory Drugs in Aqueous Sample Using Magnetic Nanoparticles Modified with Cetyltrimethylammonium Bromide by High Performance Liquid Chromatography

**DOI:** 10.1155/2014/127835

**Published:** 2014-06-01

**Authors:** Malihe Khoeini Sharifabadi, Mohammad Saber-Tehrani, Syed Waqif Husain, Ali Mehdinia, Parviz Aberoomand-Azar

**Affiliations:** ^1^Department of Chemistry, Science and Research Branch, Islamic Azad University, Poonak Hesarak, Tehran, Iran; ^2^Department of Marine Science, Iranian National Institute for Oceanography, Tehran, Iran

## Abstract

A simple and sensitive solid-phase extraction method for separation and preconcentration of trace amount of four nonsteroidal anti-inflammatory drugs (naproxen, indomethacin, diclofenac, and ibuprofen) using Fe_3_O_4_ magnetic nanoparticles modified with cetyltrimethylammonium bromide has been developed. For this purpose, the surface of MNP_s_ was modified with cetyltrimethylammonium bromide (CTAB) as a cationic surfactant. Effects of different parameters influencing the extraction efficiency of drugs including the pH, amount of salt, shaking time, eluent type, the volume of solvent, amount of adsorbent, sample volume, and the time of desorption were investigated and optimized. Methanol has been used as desorption solvent and the extracts were analysed on a reversed-phase octadecyl silica column using 0.02 M phosphate-buffer (pH = 6.02) acetonitrile (65 : 35 *v/v*) as the mobile phase and the effluents were measured at 202 nm with ultraviolet detector. The relative standard deviation (RSD%) of the method was investigated at three concentrations (25, 50, and 200 ng/mL) and was in the range of 3.98–9.83% (*n* = 6) for 50 ng/mL. The calibration curves obtained for studied drugs show reasonable linearity (*R* ^2^ > 0.99) and the limit of detection (LOD_s_) ranged between 2 and 7 ng/mL. Finally, the proposed method has been effectively employed in extraction and determination of the drugs in biological and environmental samples.

## 1. Introduction

Nonsteroidal anti-inflammatory drugs (NSAID_S_) with analgesic and antipyretic properties are used as the first-choice agents in the treatment of the allergies and reducing pain in human and veterinary medicine [1]. Recently some NSAID_S_ have been used in cancer chemotherapy and chemoprevention. In addition to the antitumor activity of NSAID_S_ as single agents, there is interest in the effects of combination chemotherapy with NSAID_S_ [2]. A review of the literature shows that naproxen, indomethacin, sodium diclofenac, and ibuprofen are often the acidic drugs (pKa = 4.2, pKa = 4.5, pKa = 4.2, and pKa = 5.2) and the residues of these compounds can enter the environment from different ways: during their manufacture, during the disposal of unused or expired drugs, and through human and animal excretions [3, 4]. Although the concentrations of these drugs are relatively low in water (ngL^−1^ to *μ*gL^−1^), continuous release and chronic exposure to these substances, because of their toxicity, can affect the intestinal, hematopoietic, and renal systems which can be a harmful to human health [5]. Therefore, development of a simple and robust method for the determination of these drugs in urine and wastewater is necessary for toxicological and therapeutic purposes. Because of their low concentration in complex matrix, different methods are used to separate and preconcentrate the analyte prior to its determination. Different combined methods have been reported for the determination of NSAID_S_ such as solid phase microextraction-high performance liquid chromatography (SPME-HPLC) [6], solid phase extraction-liquid chromatography (SPE-LC) [7, 8], solid phase extraction-gas chromatography-tandem mass (SPE-GC-MS/MS) [9], hollow fiber liquid phase microextraction (HFLPME)-HPLC [10], and molecular imprinting solid phase microextraction- (MISPME-) HPLC [11].

SPE is the most popular preconcentration method, because of high extraction efficiency, low consumption of organic solvent, low extraction time, and easy operation. Recently nanometer size particles (NP_S_) have gained rapid and substantial progress and have significantly impacted on sample extraction [12–14]. Among different kinds of MNP_S_, mainly including Fe_3_O_4_, nanoparticles were used as solid phase extraction (SPE) sorbents for preconcentration of several organic and inorganic compounds [15–18]; because of their unique size and superparamagnetic property, these particles have an interesting advanced composite material.

Magnetic nanoparticles offer many advantages over the traditional sorbents. They have very large surface area, highly active surface sites, and a short diffusion route. These particles tagged to the target can be removed from a matrix quickly by applying a magnetic field and do not agglomerate after removal of the field and can be reused or recycled easily; however, these nanometer sized metal oxides are not target-selective; therefore, overcoming this limitation modification of these magnetic nanoparticles is necessary [19–22]. Hemimicelles and admicelles are formed by the adsorption of ionic surfactants on surface of mineral oxides such as alumina, silica, titanium dioxide, and iron oxides [23] and have recently been employed as useful sorbent for the SPE of some organic compounds [24]. Few SPE methods based on surfactant-coated Fe_3_O_4_NP_s_ have been reported [25]. We report here a fast and selective method based on magnetic nanoparticles modified by CTAB for the extraction and determination of residual four nonsteroidal anti-inflammatory drugs in aqueous solution that so far has not been reported for these drugs with this method.

## 2. Experimental

### 2.1. Instruments, Reagents, and Materials

All reagents used were of analytical-reagent grade and all aqueous solutions were prepared using doubly distilled deionized water. The water was purified on a Mili-Q-ultrapure water purification system purchased from Milipore (Milford, MA,USA). Ferric chloride (FeCl_3_·6H_2_O), ferrous chloride (FeCl_2_·4H_2_O), cetyltrimethylammonium bromide (CTAB), methanol, acetonitrile, ethanol, hydrochloric acid, potassium dihydrogen phosphate, and sodium hydroxide were obtained from Merck, Germany. Naproxen (NAP), indomethacin (INDO), diclofenac (DICLO), and ibuprofen (IBU) were obtained from Daroupakhsh Drug Company (Tehran, Iran). Wastewater samples were obtained from Daroupakhsh Drug Company, and the fresh urine samples were obtained from different women patients who had or had not taken these drugs and stored at 4°C until analysis.

For magnetic separations a strong magnet of NdFeB (10 × 5 × 4 cm) was used. A HPLC (Waters, USA) system equipped with a 515 (Waters, USA) pump and a Photo Diode Array-996 detector was used. The sample injection volume was set to 20 *μ*L. A pH meter model-713 from Metrohm, Swiss, was used. The Vortex from IKA (USA), ultrasonic, was from Bandeline Sonorex (USA), transmission electron microscopy (TEM) was from Philips EM208 (Voltage 100 KV), and a centrifuge model Celements GS-200 (USA) was used.

### 2.2. HPLC Analysis and Characterization

The stationary-phase column was C_18_, Luna (5 *μ*m–250 × 4.6 mm) from Phenomenex (USA). A mixture of 0.02 M phosphate buffer (pH = 6.02) and acetonitrile (65 : 35* v/v*) was used as mobile phase and its flow rate was set at 1 mL min^−1^. The oven temperature was set at 25°C and the detection was made at the wavelength of 202 nm. The retention time for methanol as a solvent was 3 min, and retention times for NAP, INDO, DICLO, and IBU were 7.4, 15.9, 17.4, and 19.4 min, respectively.

### 2.3. Preparation of Standard Solutions and Real Samples

Stock solutions of NAP, INDO, DICLO, and IBU were prepared in methanol + phosphate buffer (pH = 7.2) (50 : 50* v/v*) and stored in dark glass/bottles at 4°C. Under these conditions, they were stable at least for one month. Working solutions (10 mg·L^−1^ naproxen, indomethacin, sodium diclofenac, and ibuprofen) were prepared daily by appropriate dilution of the corresponding stock solution in the same solvent. Urine samples were stored at 4°C until analysis. The pH of the urine samples was adjusted to 12.0 by drop wise addition of NaOH (6 M) solution. The urine samples were centrifuged at 3000 rpm for 20 min at room temperature and the supernatant was diluted (1 : 10) with deionized water. The dilution of the urine could suppress the matrix and prevent the contamination of the sorbents. Wastewater samples were collected and filtered with Millipore filter before extraction. The calibration curve for each drug was achieved by simple linear regression of each drug's peak area versus its concentration, and the concentrations of analytes in real samples were calculated on the basis of calibration curves.

### 2.4. Synthesis and Characterization of Fe_3_O_4_NP_s_

**Figure 1:**
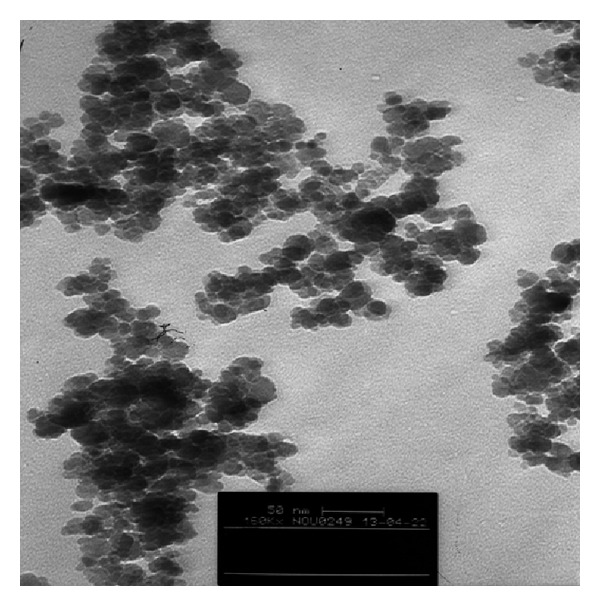
The TEM image of the synthesized MNP_s_, magnification 160000.

Fe_3_O_4_ nanoparticles were prepared by the chemical coprecipitation method as follows: 10.4 g of FeCl_3_·6H_2_O, 4 g of FeCl_2_·4H_2_O, and 1.7 mL of HCl (12 mol·L^−1^) were dissolved in 50 mL of deionized and degassed water with ultrasonic to prepare a stock solution. 500 mL of 1.5 mol·L^−1^ NaOH solution was heated to 80°C in a beaker (degassed with ultrasonic), the stock solution was added dropwise during 30 min under nitrogen gas protection, and vigorous stirring was done by a stirrer (1000 rpm) to prevent the oxidation of Fe^2+^ ions [26]. During the whole process, the temperature of the solution was maintained at 80°C and nitrogen gas was used to prevent the intrusion of oxygen. After completion of the reaction, the obtained Fe_3_O_4_NP_s_ precipitate was separated from the reaction medium by magnetic field and washed with 500 mL deionized water four times. Finally, the obtained MNP_s_ were resuspended in 500 mL of the degassed deionized water. The pH of obtained suspension was 11.0 and the concentration of the generated MNP_s_ in suspension was estimated to be about 10 mg·mL^−1^. The obtained MNP_s_ were stable up to one month. The synthesized MNP_s_ were characterized using TEM as shown in Figure 1. As it can be seen the particles diameters are lower than 50 nm.

### 2.5. Extraction Procedure

Optimization studies were carried out according to the following procedure: by the addition of appropriate volume of the drug's stock solution in 20 mL of distilled water, the aqueous solution of each drug (100 ng/mL) was prepared and then 0.75 mL of the MNP suspension (containing 10 mg of Fe_3_O_4_NP_s_) was added to the drug's solution and the pH was adjusted to 8.5. Then, 0.5 mL of the 10 mg·mL^−1^ CTAB was added and the mixture was shaken for 5 min to enhance the drug's adsorption efficiency and then by use of a strong magnet Fe_3_O_4_NP_s_ placed at the bottom of the beaker was separated quickly from sample solution. The magnet was removed and the supernatant water was decanted. Finally the drugs were desorbed with 500 *μ*L methanol from MNP_s_. Calculation of ER% showed that desorption of drugs was completed during 30 s in ultrasonic bath and 30 s in vortex. The magnet was used again to settle the MNP_s_ and the eluent was decanted into a microtube; then, 20 *μ*L of the solution was injected into the HPLC instrument for analysis. All the experiments were carried out at the room temperature. The preconcentration factor (PF) and extraction recovery (ER) of the drugs were calculated by the following equations:

(1) $$\begin{array}{c} \text{PF}=\frac{C_{E,\text{final}}}{C_{s,\text{initial}}}\times 100,\\ \text{ER}\%=\frac{n_{E}}{n_{S}}\times 100=\left(\frac{C_{E,\text{final}}\times V_{E,\text{final}}}{C_{s,\text{initial}}\times V_{s,\text{initial}}}\right)\times 100,\\ \text{ER}\%=\left(\frac{V_{E,\text{final}}}{V_{s,\text{initial}}}\right)\times \text{PF}\times 100, \end{array}$$

where  *C*_*E*,final_ and  *C*_*S*,initial_ are the final and initial concentrations of the drug in the eluent and the sample, respectively. *C*_*E*,final_ of the extracted drug was calculated from the calibration curve. *V*_*S*_ and *V*_*E*_ are the volumes of the sample solution and eluent, respectively.

## 3. Result and Discussion

### 3.1. Effect of pH

**Figure 2:**
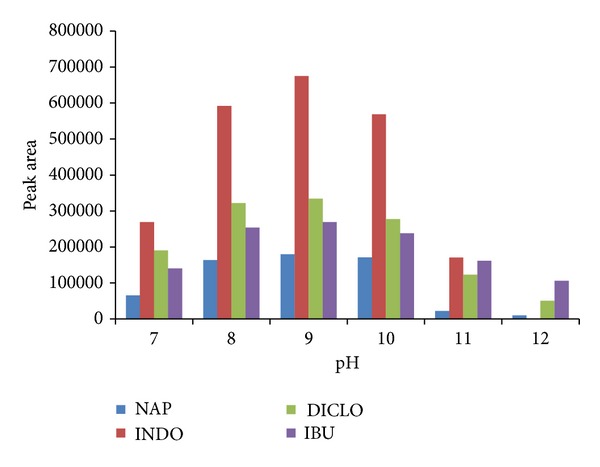
Adsorption efficiency of CTAB-coated MNP_s_ as a function of samples pH.

In order to obtain the highest recovery the effect of different parameters on the performance of the method was investigated. The charge density of mineral oxide surface is a main factor affecting the adsorption of analytes and it varies with pH. Thus, pH is a very important parameter for the adsorption of target compounds. The isoelectric point at pH = 6.5 (pH_zpc_) for the Fe_3_O_4_ nanoparticles was reported previously [27]. At the pH higher than pH_zpc_, the negative charge density on the surface of the Fe_3_O_4_NP_s_ is increased. As a result, the adsorption of CTAB as a cationic surfactant on the surface is also increased; therefore, extraction efficiency is increased. The effect of pH on adsorption performance of drugs was studied over a pH range of 7–12 for CTAB-coated MNP_s_. The adsorption performance of drugs are illustrated in Figure 2. The results indicate that maximum adsorption takes place at pH = 8.5.

### 3.2. Influence of CTAB Concentration

**Figure 3:**
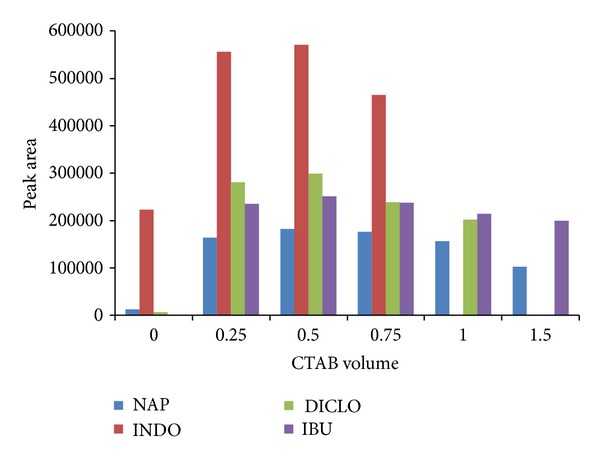
The adsorption performance of nonsteroidal anti-inflammatory drugs as a function of the amount of CTAB added.

The outer surface of hemimicelles is hydrophobic whereas that of admicelles is ionic and provides different mechanisms for retention of organic compounds which are suitable in the SPE method. In mixed hemimicelles phase, both hemimicells and micelles are formed on the surface of mineral oxides and the desorption is driven by both hydrophobic interactions and electrostatic attraction. Surfactant can form different structure on surface of metal oxides. The influence of surfactant content was studied by adding different amounts of cationic surfactant.  Figure 3 depicts the adsorption performance of drugs as a function of the amount of added CTAB. At low concentration of CTAB, the adsorption performance of the drugs on to the surface of MNP_s_ is low. In contrast, by increasing CTAB concentration, the formation of micelles in the bulk solution takes place. Therefore, when surfactant concentration was above the critical micelle concentration (CMC), the adsorption of analytes decreased gradually. Increase in the adsorption performance can be explained by the gradual formation of hydrophobic hemimicelles on the surface of MNP_s_, which can increase adsorption performance of the drugs; these results are concurrent with reported literature data [28, 29]. Hemimicelles consisting of a monolayer of surfactant is adsorbed head down on a positively charged surface when 0.5 mL of CTAB with concentration 10 mg·mL^−1^ was used.

### 3.3. Salt Effect

It is reported that the addition of salt to the samples has been beneficial for the extraction efficiency of many compounds in SPME [30]. Therefore, effect of salt addition in the range 0–10% (*w/v*) was studied on adsorption efficiency of the drugs. The results indicated that the maximum adsorption was obtained when NaCl was not used. That can be explained by the thickness of CTAB adsorption layer on the surface of MNP_s_ which led to decrease in mixed hemimicelles layer formed. Hence, no salt was added in the subsequent experiments.

### 3.4. Stirring and Magnetic Separation

In order to obtain maximum extraction efficiency, effect of the extraction time was investigated in the range of 1–10 min. Results reveal that a rapid extraction occurs in about 5 min. Then, a time of 5 min was chosen for further studies. The high surface area of MNP_s_ along with homogenous distribution of the nanosorbent throughout the sample could be the possible reason for achieving such fast extraction. Therefore, analysis time is shortened greatly compared with the traditional flow-or-batch type SPE.

### 3.5. Effect of Sample Volume

In order to obtain a higher enrichment factor, a larger volume of sample solution is required. Thus, the extraction of 100 ppb of drugs from different volumes of the water samples ranging from 20 to 80 mL was investigated. It was showed that the best quantitative recovery was obtained when the sample volume was 20 mL. The larger volume of sample leads to the analyte loss from the sorbent surface. Hence, 20 mL of sample volume was selected as the ideal volume for trace analysis of drugs in water samples.

### 3.6. Effect of the Amount of Nanoparticle Sorbent

Compared to traditional sorbents, nanoparticle sorbents have higher surface areas. Thus, satisfactory results can be obtained with lower amounts of nanoparticle sorbents. In order to study the effect of the adsorbent, different amounts of nanosorbents (0.5–2 mL of 10 mg/mL of Fe_3_O_4_NP_s_) were added to 20 mL of the sample solution. The obtained results showed that by increasing the amount of Fe_3_O_4_NP_s_ that are modified with CTAB, extraction efficiency was increased slowly due to the increasing of accessible sites; therefore, 0.75 mL of the Fe_3_O_4_NP_s_ was selected for all subsequent experiments.

### 3.7. Desorption Condition and Desorption Time

Organic solvents are known to disrupt mixed hemimicelles structures. Therefore, to find the best eluent, different organic solvents (methanol; acetonitrile; ethanol) were tested. The maximum signal was observed when methanol was used in comparison with other solvents for desorption of these drugs from the surface of the CTAB-Coated-Fe_3_O_4_NP_s_. The effect of the volume of the eluent was also tested (200–1000 *μ*L). The best results were obtained with 500 *μ*L of methanol. Effect of time of ultrasonic and shaking were also investigated in the range of 10–60 s that 20 s was found as best desorption time.

### 3.8. Optimization of Measurement Conditions

The analytical features of the proposed method such as linear range of calibration curve (correlation coefficient), preconcentration factor, limit of detection (LOD), and extraction recovery were also examined. The results are summarized in Table 1. It can be seen that the present method has high sensitivity, wide linear ranging with three replicate measurements for each point, and good method precision. The limits of detection were calculated by using signal to noise ratio of 3. It was found that preconcentration factors are in the range of (36.70–38.49) (*V*_sample_ = 20 mL) and the extraction recovery percentage values are in the range of (91.76–96.24)%. The LOD_s_ were obtained at the range of (2–7) ng·mL^−1^. The relative standard deviation (R.S.D) for the determination of 25, 50, and 200 ng·mL^−1^ of drugs were fine and are recorded in Table 2 (*n* = 6). A comparison of the features of present developed method with other reported methods for extraction and determination of nonsteroidal anti-inflammatory drugs is recorded in Table 4 which clearly shows the analytical superiority of this present method.

### 3.9. Analysis of the NSAID_S_ from Human Urine and Wastewater Samples

Owing to the importance of analysis of drugs in biological samples and wastewater in pharmaceutical company the proposed method was applied to determine the concentration of the NSAID_S_ in urine and wastewater samples. Initially the wastewater samples were spiked with four drugs at different concentrations and the extraction was performed under optimized condition. In urine sample, in order to reduce the matrix effect, pH was adjusted at 12.0 by adding NaOH; then, they were diluted tenfold with deionized water. In the next step each real sample was extracted under optimal conditions by the proposed procedure. The obtained results are recorded in Table 3. The results showed that no analyte in the nonspiked real samples was found.

**Figure 4:**
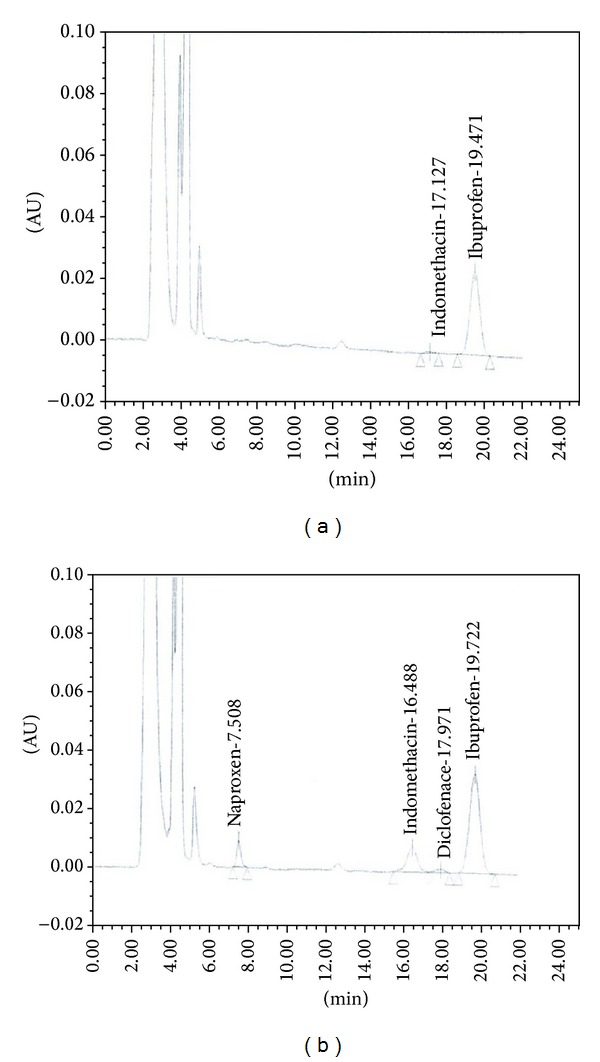
Chromatograms for nonsteroidal anti-inflammatory drugs (a) in a patient urine sample and (b) in a spike patient urine sample at 100 *μ*gL^−1^.

**Figure 5:**
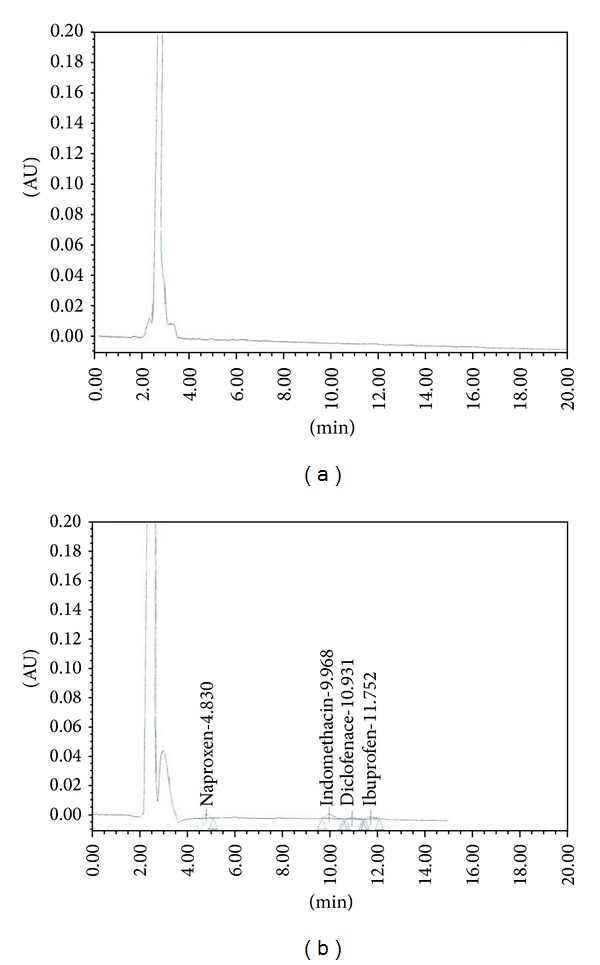
Chromatograms for nonsteroidal anti-inflammatory drugs (a) in an outlet wastewater and (b) in a spike outlet wastewater sample at 25 *μ*g L^−1^.

The chromatograms of the patient urine sample are shown in Figures 4(a) and 4(b). The chromatograms of the outlet wastewater samples with 25 ng/mL concentration level of the target drugs are shown in Figures 5(a) and 5(b). These chromatograms reveal a good cleanup of the proposed method to determine drug concentration in the urine samples. Also, the obtained results for the spike urine samples are in reasonable agreement with the respective values.

## 4. Conclusion 

A new rapid and sensitive method for preconcentration and determination of residual of four (NSAID_s_) drugs in wastewater and urine samples has been developed based on use of superparamagnetic CTAB-coated magnetic nanoparticles. It is clear that in comparison to other reported methods the present developed method has striking advantages such as high sensitivity, consuming low amount of organic solvent (500 *μ*L), short analysis time (17 min), and employing small amount of nanoparticles sorbent due to their higher surface area. Further no centrifugation or filtration required for removal of magnetic CTAB-coated nanoparticles. These advantages together with the inherent high sensitivity and selectivity of HPLC make this method a reliable and robust methodology for trace analysis of organic and biological species in a variety of samples.

## Figures and Tables

**Table 1:** Analytical characteristics of SPE based on CATB-coated MNP_s_ for the determination of nonsteroidal anti-inflammatory drugs.

Analyte	LOD (ngmL^−1^)^a^	*R* ^2^	Regression equation	LDR (ngmL^−1^)^b^	PF	ER%
DICLO	7	0.997	*A* = 1.042*C* (*μ*gL^−1^) + 2.537	10–200	36.70	91.76
INDO	2	0.996	*A* = 3.939*C* (*μ*gL^−1^) − 9.662	2.5–400	38.49	96.24
IBU	3	0.999	*A* = 2.128*C* (*μ*gL^−1^) − 6.848	5–400	37.74	94.36
NAP	2	0.999	*A* = 1.514*C* (*μ*gL^−1^) − 1.325	10–400	38.04	95.11

^a^Limit of detection.

^b^Linear dynamic range.

**Table 2:** Average relative standard deviation (RSD) for spiked drugs (*n* = 6).

Analyte	25 ng·mL^−1^	50 ng·mL^−1^	200 ng·mL^−1^
DICLO	10.94%	9.83%	8.16%
INDO	7.02%	6.61%	4.45%
IBU	5.86%	3.98%	3.85%
NAP	5.53%	4.11%	3.73%

**Table 3:** Determination of the INDO in wastewater and INDO, IBU in urine samples.

Sample	INDo	IBu
(1) Inlet of wastewater		
Initial concentration (*μ*g·L^−1^)	1.91	—
RSD%	6.41	—
(2) Inlet of wastewater (25 *μ*g·L^−1^ of the drug was added)		
Found	25.83	—
RSD%	5.24	—
Preconcentration factor	38.39	—
(3) Outlet of wastewater		
Initial concentration (*μ*g·L^−1^)	—	
(4) Outlet of wastewater (25 *μ*g·L^−1^ of the drug was added)		
Found	24.71	—
RSD%	4.93	—
Preconcentration factor	39.53	—
(5) Urine (patient) (100 *μ*g·L^−1^ of the drugs was added)		
Initial concentration (*μ*g·L^−1^)	10.10	689.89
Found	107.61	751.37
RSD %	4.11	1.70
Preconcentration factor	39.09	38.05
(6) Urine (100 *μ*g/Lit of the drug was added)		
Initial concentration	—	—
Found	100.07	97.45
RSD%	4.66	3.93
Preconcentration factor	40.02	38.98

**Table 4:** Comparison of the proposed method with other analytical methods for the determination of different nonsteroidal anti-inflammatory drugs in various samples.

Analytical technique	Matrix	Linear range	*R* ^2^	LOD	ER%	RSD%	Reference
HFLPME-HPLC (Ibu-Diclo)	Urine	(135–10000) ng/mL	>0.99	(41–53) ngmL^−1^	99%		[9]
SPME-HPLC (Ibu)	Urine	(5–50) *μ*gmL^−1^	>0.98	5 *μ*gmL^−1^	(3.7–5.7) %	<13.4	[6]
Supra molecular SPE-LC (Nap-Ibu)	Sewage	Ibu (0.2–750) ng Nap (0.02–250) ng	>0.99 >0.99	0.8 ngmL^−1^ 9 ngmL^−1^	(93–101) %	(2–9) %	[8]
SPE-LC (Diclo-Indo)	Urine	(0.02–1.0) *μ*gmL^−1^	>0.99	0.007–0.035 *μ*gL^−1^	(85-85) %	Diclo (0.95–9.8) % Indo (0.62–8.7) %	[7]
MSPE-HPLC (NSAIDs)	Urine and sewage	(7–200) ng/mL	>0.99	(2–7) ng/mL	(91.76–96.24) %	(3.98–9.83) %	This work

SPME: solid phase micro extraction; SPE: solid phase extraction; HFLPME: hollow fiber liquid phase microextraction; MNP_s_: magnetic nanoparticles.
